# Dominance of *Endozoicomonas* bacteria throughout coral bleaching and mortality suggests structural inflexibility of the *Pocillopora verrucosa* microbiome

**DOI:** 10.1002/ece3.3830

**Published:** 2018-01-25

**Authors:** Claudia Pogoreutz, Nils Rädecker, Anny Cárdenas, Astrid Gärdes, Christian Wild, Christian R. Voolstra

**Affiliations:** ^1^ Red Sea Research Center, Biological and Environment Science and Engineering Division (BESE) King Abdullah University of Science and Technology (KAUST) Thuwal Saudi Arabia; ^2^ Marine Ecology Group Faculty of Biology and Chemistry University of Bremen Bremen Germany; ^3^ Coral Reef Ecology Group Leibniz Center for Tropical Marine Research (ZMT) Bremen Germany; ^4^ Tropical Marine Microbiology Group Leibniz Center for Tropical Marine Research (ZMT) Bremen Germany

**Keywords:** coral reefs, host–microbe interaction, metaorganism, microbiome, pollution, symbiosis

## Abstract

The importance of *Symbiodinium* algal endosymbionts and a diverse suite of bacteria for coral holobiont health and functioning are widely acknowledged. Yet, we know surprisingly little about microbial community dynamics and the stability of host‐microbe associations under adverse environmental conditions. To gain insight into the stability of coral host‐microbe associations and holobiont structure, we assessed changes in the community structure of *Symbiodinium* and bacteria associated with the coral *Pocillopora verrucosa* under excess organic nutrient conditions. *Pocillopora*‐associated microbial communities were monitored over 14 days in two independent experiments. We assessed the effect of excess dissolved organic nitrogen (DON) and excess dissolved organic carbon (DOC). Exposure to excess nutrients rapidly affected coral health, resulting in two distinct stress phenotypes: coral bleaching under excess DOC and severe tissue sloughing (>90% tissue loss resulting in host mortality) under excess DON. These phenotypes were accompanied by structural changes in the *Symbiodinium* community. In contrast, the associated bacterial community remained remarkably stable and was dominated by two *Endozoicomonas* phylotypes, comprising on average 90% of 16S rRNA gene sequences. This dominance of *Endozoicomonas* even under conditions of coral bleaching and mortality suggests the bacterial community of *P. verrucosa* may be rather inflexible and thereby unable to respond or acclimatize to rapid changes in the environment, contrary to what was previously observed in other corals. In this light, our results suggest that coral holobionts might occupy structural landscapes ranging from a highly flexible to a rather inflexible composition with consequences for their ability to respond to environmental change.

## 1 INTRODUCTION

Anthropogenic environmental change has caused global degradation and loss of coral reef cover at an unprecedented scale over the last decades (Hughes et al., 2017). Slowing or even reverting coral reef decline requires a detailed understanding of the mechanisms and drivers underpinning the health of their main ecosystem engineers: reef‐building corals. Corals are metaorganisms consisting of the coral host, dinoflagellate algae of the genus *Symbiodinium*, and a multitude of other microbes including bacteria, archaea, viruses, and protists (Rohwer, Seguritan, Azam, & Knowlton, 2002). Hence, the biology and performance of such holobionts are, at least in part, governed by the interactions of their individual members (Rosenberg, Koren, Reshef, Efrony, & Zilber‐Rosenberg, 2007). The functional importance and long‐term stability of the nutrient‐exchange symbiosis between corals and *Symbiodinium* is well documented (Falkowski, Dubinsky, Muscatine, & Mccloskey, 1993; Falkowski, Dubinsky, Muscatine, & Porter, 1984; Muscatine & Porter, 1977). In contrast, despite the putative importance of other coral‐associated microbes, we still know comparatively little about how stable these associations are (Neave, Rachmawati, et al., 2017; Roder, Arif, Daniels, Weil, & Voolstra, 2014; Röthig, Ochsenkühn, Roik, Merwe, & Voolstra, 2016; Sunagawa et al., 2009; Vega Thurber et al., 2009; Wegley, Edwards, Rodriguez‐Brito, Liu, & Rohwer, 2007). In particular, coral‐associated bacteria are putative important members of the coral holobiont, as they may provide functions associated with pathogen defense, metabolic cycling (including nitrogen, carbon, and sulfur cycling), and, importantly, may contribute to holobiont resilience to environmental stress (Krediet, Ritchie, Paul, & Teplitski, 2013; Pogoreutz, Rädecker, Cárdenas, Gärdes, Wild, et al., 2017; Rädecker, Pogoreutz, Voolstra, Wiedenmann, & Wild, 2015; Raina, Dinsdale, Willis, & Bourne, 2010; Rosenberg et al., 2007; Shnit‐Orland & Kushmaro, 2009; Wegley et al., 2007; Ziegler, Seneca, Yum, Palumbi, & Voolstra, 2017).

Given the likely critical contribution of bacteria to coral holobiont function, changes in the identity and abundance of associated bacteria may allow for rapid adaptation to environmental change (Reshef, Koren, Loya, Zilber‐Rosenberg, & Rosenberg, 2006; Rosenberg et al., 2007; Theis et al., 2016; Torda et al., 2017). Consequently, a dynamic relationship between associated microorganisms and environmental conditions is assumed that selects for the most beneficial host microbiome (Reshef et al., 2006). Thereby, diversity and function of microbes need to be considered when assessing acclimatization and adaptation of corals and the ecosystems they shape (Ainsworth & Gates, 2016; Jessen et al., 2013; Pogoreutz, Rädecker, Cárdenas, Gärdes, Wild, et al., 2017; Röthig et al., 2016; Ziegler, Seneca, et al., 2017). Indeed, previous studies found that stressed coral holobionts exhibited changes in the bacterial community structure (Jessen et al., 2013; Röthig et al., 2016; Ziegler, Seneca, et al., 2017), although it is unclear whether changes in host performance and bacterial community structure constitute a parallel response or are functionally linked (Pogoreutz, Rädecker, Cárdenas, Gärdes, Wild, et al., 2017; Ziegler, Seneca, et al., 2017). Bacterial community shifts were also observed in severely stressed, diseased, and bleached corals where they are commonly associated with an increase in opportunistic and potentially pathogenic bacteria coupled with physiological impairment of the holobiont (Bourne, Iida, Uthicke, & Smith‐Keune, 2008; Cárdenas, Rodriguez‐R, Pizarro, Cadavid, & Arévalo‐Ferro, 2012; Cooney, Pantos, Le Tissier, Barer, & Bythell, 2002; Roder, Bayer, Aranda, Kruse, & Voolstra, 2015; Roder, Arif, Bayer, et al., 2014; Roder, Arif, Daniels, et al., 2014; Vega Thurber et al., 2009). In these cases, changes in the coral‐associated bacterial community composition were interpreted as detrimental. Accordingly, changes in bacterial community structure or activity may be either beneficial or deleterious, depending on the environmental context (Ainsworth & Gates, 2016; Pogoreutz, Rädecker, Cárdenas, Gärdes, Voolstra, et al., 2017; Pogoreutz, Rädecker, Cárdenas, Gärdes, Wild, et al., 2017; Rädecker et al., 2015, 2017; Santos et al., 2014).

Although putative core members (Ainsworth et al., 2015; Hernandez‐Agreda, Gates, & Ainsworth, 2017; Hernandez‐Agreda, Leggat, Bongaerts, & Ainsworth, 2016; Neave, Rachmawati, et al., 2017) and temporally stable versus more sporadic bacterial taxa (Hester, Barott, Nulton, Vermeij, & Rohwer, 2016) of coral microbiomes were previously identified, the common denominator of coral‐associated microbiomes may ultimately be its flexibility. The notion of flexible bacterial associations, however, has never been systematically assessed. Further, it remains unclear whether all corals possess flexible bacterial microbiomes. Here we sought to investigate the flexibility of microbial association of the common Indo‐Pacific coral *Pocillopora verrucosa*, previously suggested to display a limited acclimatization potential (Pogoreutz, Rädecker, Cárdenas, Gärdes, Voolstra, et al., 2017; Pogoreutz, Rädecker, Cárdenas, Gärdes, Wild, et al., 2017; Sawall et al., 2015; Ziegler, Roder, Büchel, & Voolstra, 2014). We approached this by exposing the *P. verrucosa* holobiont to different excess dissolved organic nutrient treatments. Specifically, we integrated microbial community data from the here‐conducted experiment assessing the effects of excess dissolved organic nitrogen (DON; 40‐ to 50‐fold enrichment compared to ambient conditions) and complemented these data with a previously published companion experiment exposing *P. verrucosa* holobionts to excess labile dissolved organic carbon (DOC; >10‐fold enrichment; Pogoreutz, Rädecker, Cárdenas, Gärdes, Wild, et al., 2017). In these two independent 14‐day experimental treatments, corals exhibited bleaching and mortality reflected by progressed tissue sloughing, respectively. The composition and concentration of the excess DOC treatment were selected to mimic sewage input (Huang, Li, & Gu, 2010) and exudates of the reef macroalga *Halimeda* (Nelson et al., 2013), which share a similar pool of the most abundant oligosaccharides as used here. The excess DOC and excess DON conditions in the present study constitute severe stress scenarios not representative of natural, healthy reef conditions (Pogoreutz, Rädecker, Cárdenas, Gärdes, Wild, et al., 2017), but rather reflect conditions of degraded coastal ecosystems impacted by anthropogenic activity (Kline, Kuntz, Breitbart, Knowlton, & Rohwer, 2006; Peña‐García, Ladwig, Turki, & Mudarris, 2014). Excess organic matter can cause rapid and dramatic compositional and metabolic changes in bacterial communities (Cárdenas et al., 2017; Haas et al., 2016) and can affect the physiology of coral holobionts (Kline et al., 2006; Vega Thurber et al., 2009, 2014). As the uptake and cycling of organic nutrients involve all holobiont members, choosing an approach to assay *Symbiodinium* and bacterial community dynamics allows to identify rapid responses associated with and potentially involved in holobiont resilience and/or breakdown. We assessed such responses in algal and bacterial symbionts by assessing community composition dynamics via ITS2 and 16S rRNA gene‐typing, respectively, alongside algal symbiont density and chlorophyll *a* content measurements.

## 2 MATERIALS AND METHODS

The data presented in this manuscript were collected in two independent companion experiments during November/December 2014 (excess DOC experiment) and January/February 2015 (excess DON experiment) at the wet laboratory facilities of the Coastal and Marine Resources Core Lab (CMOR) at the King Abdullah University of Science and Technology (KAUST). Part of the data presented for the excess DOC experiment were published previously in a companion paper (Pogoreutz, Rädecker, Cárdenas, Gärdes, Wild, et al., 2017); this applies to the bacterial 16S rRNA gene amplicon sequencing data, *Symbiodinium* population density data, and seawater DOC concentrations for the excess DOC experiment presented in this study. This is complemented by new data on *Symbiodinium* chlorophyll *a* content and ITS2 sequencing for the excess DOC and DON experiments, as well as bacterial 16S rRNA gene amplicon sequencing data for the DON experiment. For the present study, the specified data previously published in Pogoreutz, Rädecker, Cárdenas, Gärdes, Wild et al. (2017) along with the new data from both experiments were jointly analyzed.

### 2.1 Coral collection and husbandry

Three colonies of the brown color morph of *P. verrucosa* were collected for each of the excess nutrient experiments (i.e., a total of *n* = 6 coral colonies) from a Central Red Sea midshore reef (“Al‐Fahal” reef, N22°18′19.98″, E38°57′46.08″; Kingdom of Saudi Arabia). The colonies had an average diameter of 40 cm and were collected from 7 to 8‐m water depth. Care was taken to sample corals at least 5 m apart from each other to avoid collection of clonal colonies of *P. verrucosa* (Robitzch, Banguera‐Hinestroza, Sawall, Al‐Sofyani, & Voolstra, 2015).

Immediately after coral collection for each of the experiments (i.e., excess DOC and excess DON), coral colonies were fragmented and acclimated for a 4‐week period in aquaria at the wet laboratory facilities. For each of the experiments, the aquarium system consisted of two separate identical units, each consisting of three closed replicate experimental tanks (100 L) connected to reservoir bins (100 L) containing filtration and heating equipment. For each of the experiments, one of the units was maintained at ambient conditions (control), while the second unit was used for the respective nutrient manipulation (excess DOC or DON); the two experimental nutrient manipulations were conducted independently from each other and consecutively. Red Sea reef water was circulated in each tank, and 30% of the water was replaced on a daily basis, maintaining close to natural water parameters. Maintenance conditions were kept constant (temperature 26.9 ± 0.4°C, salinity 41.0 ± 0.8 PSU, photosynthetic active radiation ~100 quanta μmol m^−2^ s^−1^, on a 12:12‐hr daylight cycle; dissolved oxygen levels remained >6 mg/L at all times). Individual colonies were fragmented and glued to 40 × 40 mm stone tiles with a two‐part epoxy putty. After the acclimation period, replicate fragments of each of the 3 colonies in each excess nutrient experiment were redistributed among the six aquarium tanks (i.e., 100 L each and each tank contained replicates of each colony) of the two separate identical units (Figure S1). The sampling design was fully paired, except for day 14 in the excess DON treatment. Here, due to mortality of individual fragments, colony replication was *n* = 2 for day 14.

### 2.2 DOC and DON enrichment experiments

For each of the experiments (i.e., excess DOC and excess DON), three tanks per unit were used for nutrient manipulations and three tanks of the remaining unit were used as controls, that is, maintained at ambient levels (see above). In the first experiment, elevated DOC conditions were achieved in three aquarium tanks by daily additions of a monosaccharide mixture (10 mg/L; composition: (in mg/L; (D+) xylose: 3.82; (D+) glucose: 2.56; (D+) mannose: 1.39; (D+) galactose: 2.22; Pogoreutz, Rädecker, Cárdenas, Gärdes, Wild, et al., 2017). The respective contribution of each monosaccharide was based on the carbohydrate composition of sewage (Huang et al., 2010) and released exudates of the coral reef macroalga *Halimeda* after chemical hydrolysis (Nelson et al., 2013). In the second experiment, dissolved organic nitrogen (DON) levels were increased in three of the aquarium tanks of one unit by the addition of 0.05 mg/L of polymer‐coated slow release urea fertilizer pellets (Duration Urea 45, Agrium Advanced Technologies, Inc., Loveland, CO, USA) on a weekly basis. The three tanks of the remaining unit were used as controls. Urea is increasingly detected at elevated concentrations in polluted coastal waters (up to 6 μM in the vicinity of wastewater outlets in the Jeddah Metropolitan area in the otherwise oligotrophic Central Red Sea, where urea is found at concentrations of 0.2–0.5 μM; Peña‐García et al., 2014). Urea rapidly photodissociates into carbon dioxide and ammonium in aqueous solutions (Glibert, Harrison, Heil, & Seitzinger, 2006), thereby allowing a continuous co‐enrichment of dissolved organic and inorganic nitrogen. Notably, while urea is predominantly taken up by the coral animal itself (Grover, Maguer, Allemand, & Ferrier‐Pagès, 2006), ammonium is preferably taken up by algal symbionts (Grover, Reynaud‐Vaganay, & Ferrier‐Pagès, 2002). Thereby, the here applied excess nitrogen conditions ensured the co‐enrichment of both the coral animal and the associated algal symbionts.

### 2.3 Sampling and measurements

All sampling procedures and measurements were identical for both experiments. Fragments for all response parameters were sampled on day 0, 7, and 14 for the respective experiments. For the assessment of *Symbiodinium* cell density and chlorophyll *a* content, *Symbiodinium* typing, and bacterial community analyses, single fragments originating from all mother colonies were freshly collected for each treatment condition and time point and rinsed with filter‐sterilized seawater (FSW; 0.22 μm), flash‐frozen in liquid nitrogen, and stored at −80°C until further processing. Seawater samples for bacterial community analyses were collected in triplicates (1 L each) for each treatment and time point. The seawater samples were filtered through 0.22 μm, and the filters immediately frozen and stored at −80°C until further processing. Coral tissue loss was assessed by visual estimates based on photographs.

### 2.4 Seawater nutrient analysis

Water samples for the analysis of nutrients were collected at all sampling points in 30 ml triplicates for each condition. Each water sample was filtered (GFF 0.7 μm, Fisher Scientific, USA). Water samples for DOC measurements were additionally filtered through 0.45‐μm GFF filters to remove particulate organic carbon and subsequently acidified with 100 μl of 35% phosphoric acid to remove inorganic carbon content. Subsequently, all water samples were frozen at −20°C. Frozen water samples for DOC analysis were defrosted prior to analysis and then measured with an Apollo 9000 Total Organic Carbon (TOC) analyzerTM (Teledyne Instruments Tekmar, USA; for DOC experiment only). Frozen water samples for the measurement of total dissolved nitrogen (TDN; both experiments) were submitted to the Marine Chemistry Lab (University of Washington, Seattle) for analysis.

### 2.5 *Symbiodinium* cell density and chlorophyll analysis

For the assessment of *Symbiodinium* population densities and chlorophyll *a* content *in hospite*,* Symbiodinium* cells were freshly isolated from coral tissue by NaOH extraction (Zamoum & Furla, 2012). Subsamples of individual coral fragments were incubated in 1M NaOH. After 1 hr, the skeleton was removed. Suspended *Symbiodinium* cells were spun down in a bench‐top centrifuge for 5 min at 3,000 RCF, the supernatant discarded, and the *Symbiodinium* pellet resuspended in 1 ml 1 × PBS. After a second centrifugation step, the pellet was resuspended in a 10% PBS‐buffered formaldehyde solution and stored at 4°C until further processing. *Symbiodinium* density was determined using flow cytometry (BD LSRFortessa, BD Biosciences, USA). The relative cell chlorophyll *a* content of *Symbiodinium* per sample (i.e., for each coral fragment) was calculated based on the relative chlorophyll autofluorescence of *Symbiodinium* cells. For this, cells were excited at a wavelength of 488 nm and fluorescence emission was recorded at 695/40 nm.

### 2.6 DNA extraction from seawater and coral samples, PCR conditions, and sequencing

To address changes in the *Symbiodinium* community (coral‐associated) and bacterial community (seawater and coral‐associated) composition, we sequenced the ribosomal internal transcribed spacer 2 (ITS2) and the hypervariable regions v5 and v6 of the 16S rRNA gene, respectively.

Before DNA extraction, each frozen coral fragment was transferred into a sterile zip‐lock bag. While thawing, fragments were doused with 5‐ml Qiagen AP‐1 tissue lysis buffer (Qiagen, Germany) and tissue was subsequently removed by air‐blasting on ice. The coral tissue slurry in AP‐1 was stored frozen at −80°C until further processing. DNA was extracted from the tissue slurry and seawater filters using the Qiagen DNeasy Plant Mini Kit (Qiagen, Germany) according to manufacturer's instructions. Two‐hundred microliters tissue slurry in 200‐μl AP‐1, 4‐μl RNase A stock solution (100 g/ml), and 200‐μl 0.5‐mm sterile glass beads (BioSpec, USA) were bead‐beaten at 30 Hz for 90 s with a TissueLyser II (Qiagen, Germany). Extracted DNA was quantified and quality checked using a NanoDrop 2000C spectrophotometer (Thermo Fisher Scientific, USA).

For all samples, PCR amplifications were performed in triplicates using Qiagen Multiplex PCR Kit (Qiagen, Germany) with primers containing Illumina adapters (underlined below). For *Symbiodinium* typing, we used the primers ITSintfor2 5′‐ TCGTCGGCAGCGTCAGATGTGTATAAGAGACAGGAATTGCAGAACTCCGTG‐3′ and MiSeq‐ITS2‐reverse 5′‐ GTCTCGTGGGCTCGGAGATGTGTATAAGAGACAGGGGATCCATATGCTTAAGTTCAGCGGGT‐3′ (Arif et al., 2014). To amplify the hypervariable regions v5 and v6 of the bacterial 16S rRNA gene, we used the primers 16SMiSeqF‐Andersson 5′‐TCGTCGGCAGCGTCAGATGTGTATAAGAGACAGAGGATTAGATACCCTGGTA‐3′ and 16SMiSeqR‐Andersson 5′‐GTCTCGTGGGCTCGGAGATGTGTATAAGAGACAGCRRCACGAGCTGACGAC‐3′ (Andersson et al., 2008). Individual PCRs were run using 10‐μl Qiagen Mix, 0.5 μl of each 10‐μM primer mix, 1 μl of DNA template (10 ng/μl) and RNase‐free water to adjust to a final reaction volume of 20 μl. Thermal cycling conditions for ITS2 were: 94°C for 15 min, then 35 cycles of 94°C for 30 s, 51°C for 30 s, 72°C for 30 s, followed by one cycle of 72°C for 10 min, and hold at 4°C (for ITS2 amplification); thermal cycling conditions for 16S rRNA PCRs were: 95°C for 15 min, followed by 27 cycles of 95°C for 40 s, 55°C for 40 s, 72°C for 40 s, and a final extension cycle of 72°C at 10 min. Ten microliters of each PCR product were run on an 1% agarose gel to visualize successful amplification. Sample triplicates were subsequently pooled and then purified using the Agencourt AMPure XP magnetic bead system (Beckman Coulter, Brea, CA, USA). Purified PCR products were subjected to an indexing PCR (8 cycles) to add Nextera XT indexing and sequencing adapters (Illumina, USA) according to the manufacturer's protocol. Indexed products were again purified, quantified on the BioAnalyzer (Agilent Technologies, USA) and QuBit (Quant‐IT dsDNA Broad Range Assay Kit; Invitrogen, USA), and pooled in equimolar ratios. The final pooled library was purified on a 2% agarose gel to remove primer dimer. The library was sequenced at 8pM with 10% phiX on the Illumina MiSeq, 2 × 300 bp end version 3 chemistry according to the manufacturer's specifications at the Bioscience Core Lab (KAUST, Saudi Arabia).

### 2.7 ITS2 sequencing data analysis for *Symbiodinium* community composition

Sequences from the Illumina MiSeq platform were processed with *mothur* v1.36.1n (Schloss et al., 2009). Sequences were demultiplexed, quality trimmed, preclustered (2 bp difference), and split according to barcodes. For the ITS2 amplicon analysis, sequences were processed according to the procedures detailed in Arif et al. (2014). *Symbiodinium* paired‐end ITS2 sequences were merged using the *make.contigs* command in *mothur*. Subsequently, forward and reverse primers were trimmed with CUTADAPT (Martin, 2011). Sequencing reads were quality trimmed with the *screen.seqs* command and checked for chimeric sequences with *chimera.uchime* in *mothur* (Edgar, Haas, Clemente, Quince, & Knight, 2011). Quality‐filtered sequences were collapsed with *unique.seqs*, and singletons removed with *split.abund*. This yielded a total of 4,818,958 sequences, distributed over 1,230,805 distinct sequences with an average length of 287 bp. Due to the known intragenomic variance of the ITS2 region in *Symbiodinium* (Smith, Ketchum, & Burt, 2017), we assigned unique ITS2 sequences to *Symbiodinium* clades against a custom BLAST database without further clustering into operational taxonomic units (OTUs; Arif et al., 2014; Ziegler, Arif, & Burt, 2017).

### 2.8 16S rRNA gene amplicon sequencing data analysis for bacterial community composition

Bacterial 16S rRNA gene amplicon sequences were processed according to mothur MiSeq SOP (accession date: Feb 13th 2017; Schloss et al., 2009). In brief, sequences were assembled into contigs and quality trimmed. Identical sequences (duplicates) were merged. Singletons and rare sequences (*n* < 10 over all samples) were removed. This resulted in 12,041,000 sequences distributed over 36 coral fragments [3 coral replicates × 4 conditions (control vs. DOC; control vs. DON) × 3 time points] and 36 water samples [3 seawater replicates × 4 conditions (control vs. DOC; control vs. DON) × 3 time points]. Remaining sequences were aligned against the SILVA database (release 119; Pruesse et al., 2007)) and preclustered (2 bp difference; Huse, Welch, Morrison, & Sogin, 2010). Chimeric sequences were removed using the UCHIME command (Edgar et al., 2011). Sequences assigned to chloroplasts, mitochondria, archaea, and eukaryotes were removed based on classification against the Greengenes database (release gg_13_8_99, McDonald et al., 2012). Further, kit contaminants were removed based on sequencing results of PCRs from negative controls, including *Brevibacterium casei*,* B. aureum*,* Brachybacterium* sp., *Dietzia* sp., *Pelomonas puraquae*, and *Simkania negevensis* (Salter et al., 2014). After removal of unwanted sequences, 3,576,201 sequences with an average length of 309 bp were retained for subsequent analyses, clustered into OTUs (97% similarity cutoff), and annotated against the Greengenes database (release gg_13_8_99, bootstrap = 60; McDonald et al., 2012). Bacterial community composition pie charts were created on the OTU and class level using the means of relative abundances from replicates (*n* = 3). All raw sequence data are accessible under NCBI's BioProject ID (PRJNA394597).

### 2.9 Statistical analysis

All statistical analyses of *Symbiodinium* responses and microbial alpha diversity were conducted in R v3.3.0 (R Development Core Team, 2015). *Symbiodinium* population density as well as relative abundance of clade A and D symbionts was tested for significant individual and interactive effects of treatment and time in generalized linear models (GLMs). The models were based on a Gamma distribution with best fitting link function to account for skewing of data. To illustrate significant differences between manipulations, treatment effects of individual time points were compared using unpaired Welch's unequal variances *t* test. Bacterial community compositions were compared between treatment and time points using analysis of molecular variance (AMOVA) as implemented in *mothur*. Alpha diversity indices of bacterial communities were compared between treatment and time points using two‐way analysis of variance (ANOVA), and homoscedasticity of models was confirmed using the Breush–Pagan test as implemented in the “lmtest” package (Hothorn et al., 2017).

## 3 RESULTS

### 3.1 Coral phenotypic response to excess DOC and DON

**Figure 1:**
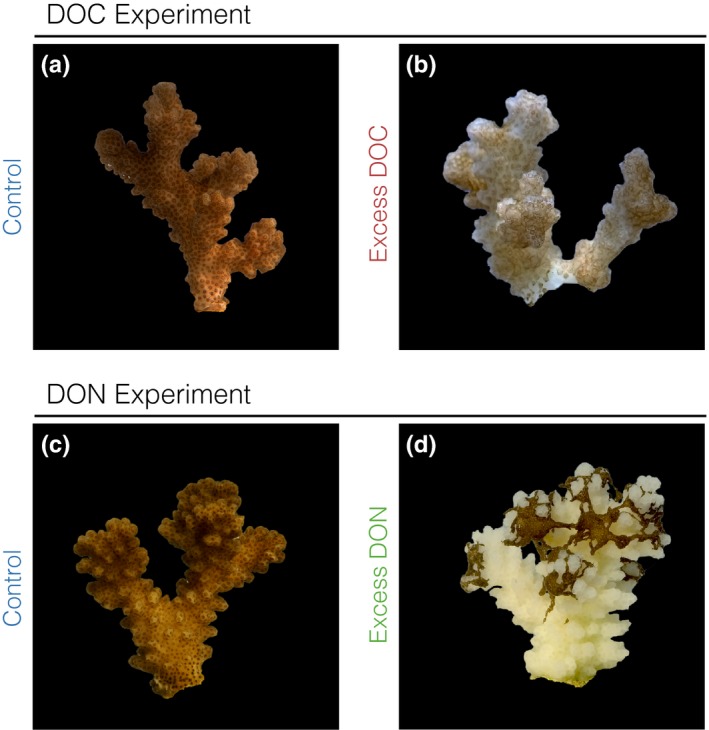
Phenotypic response of *Pocillopora verrucosa* subjected to excess dissolved organic carbon (DOC) and excess dissolved organic nitrogen (DON). (a and b) Corals exposed to excess DOC exhibited pronounced bleaching over the course of the 14‐day treatment compared to control coral colonies. (c and d) Corals exposed to excess DON exhibited a marked darkening of the tissues, even when partial mortality (>90% tissue loss) was visible (day 14)

**Figure 2:**
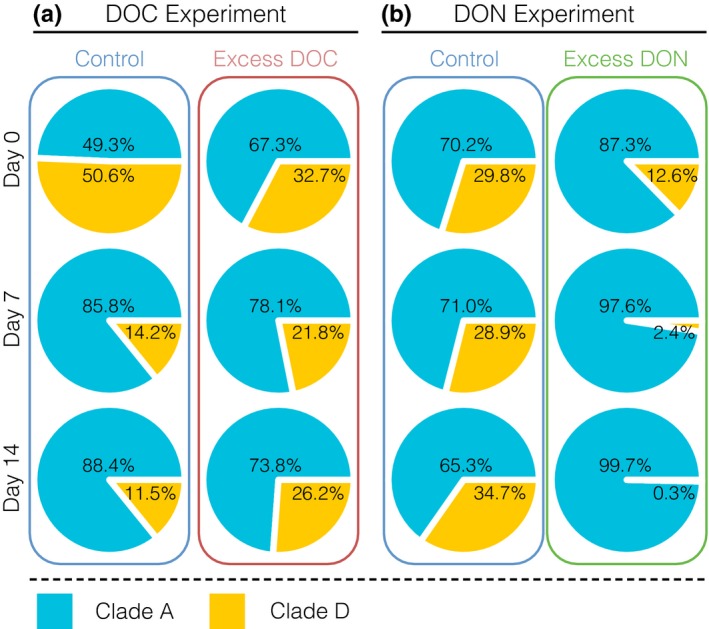
*Symbiodinium* clade composition in the coral *Pocillopora verrucosa* subjected to excess dissolved organic carbon (DOC) and excess dissolved organic nitrogen (DON) over time. (a) Excess DOC, (b) excess DON. Sequences annotated to *Symbiodinium* clades A and D made up >99% of the *Symbiodinium* population associated with *P. verrucosa*

**Figure 3:**
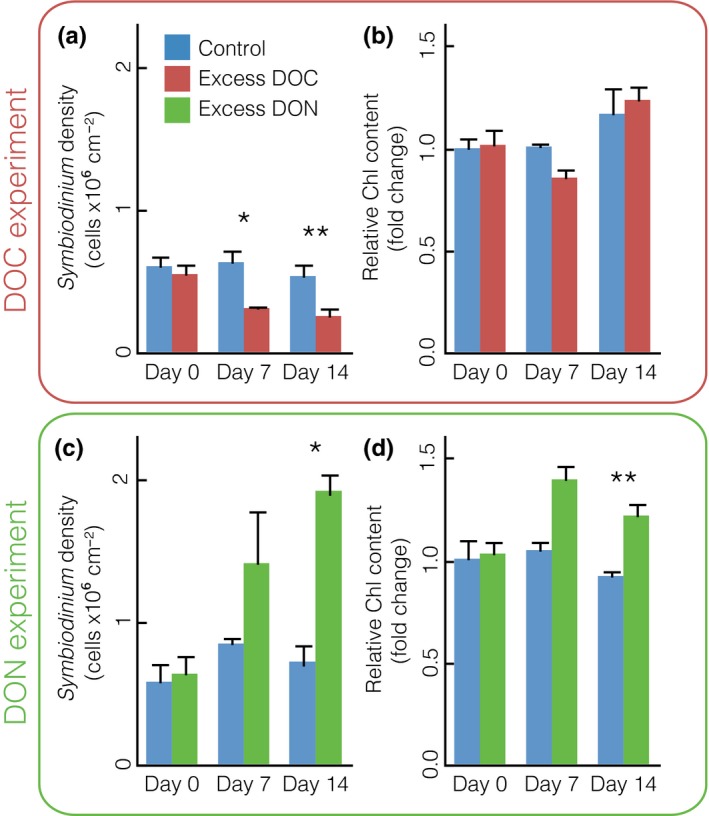
Response of *Symbiodinium* populations in the coral *Pocillopora verrucosa* subjected to excess dissolved organic carbon (DOC) and excess dissolved organic nitrogen (DON) over time. (a) Population density under excess DOC; (b) relative cell chlorophyll content under excess DOC; (c) population density under excess DON; (d) cell chlorophyll *a* content under excess DON. Asterisks indicate significant differences between treatments (*p < .05, **p < .01). *Symbiodinium* population densities under excess DOC were previously presented in Pogoreutz, Rädecker, Cárdenas, Gärdes, Wild et al. (2017)

The enrichment with excess labile dissolved organic carbon (DOC; >10‐fold enrichment) and nitrogen (DON; 40‐ to 50‐fold enrichment compared to ambient conditions) during two independent 14‐day experimental treatments resulted in severe coral stress phenotypes (for details, refer to Table S1). While the control fragments remained in a visibly healthy state, coral fragments under excess DOC exhibited a bleaching response (Figure 1; Pogoreutz, Rädecker, Cárdenas, Gärdes, Wild, et al., 2017). Conversely, all coral fragments subjected to DON initially (within 7 days of treatment) displayed visibly darkened tissue. However, minor tissue loss (<10%) became apparent after 13 days of the DON treatment, followed by progressed tissue sloughing (>90% tissue loss, resulting in mortality) of all treated fragments on day 14. Notably, at this point, remaining coral tissue from coral fragments under excess DON remained visibly darkened in comparison with the tissue of control coral fragments (Figure 1a,b). The two distinct coral stress phenotypes were accompanied by corresponding changes in *Symbiodinium* population density (50% loss of *Symbiodinium* cells under excess DOC, GLM, $\chi_{\left(5,n=18\right)}^{2}$ = 35.407; *p *<* *.001, Figure 2b; 108% increase in *Symbiodinium* cells under excess DON, GLM, $\chi_{\left(5,n=18\right)}^{2}$ = 34.791, *p *<* *.0001; Figure 3a,c). Under excess DOC, no change in *Symbiodinium* relative chlorophyll content was observed. In contrast, under excess DON a 30% increase in relative chlorophyll *a* content was measured at 7 and 14 days (GLM, $\chi_{\left(5,n=18\right)}^{2}$ = 36.895, *p* < .001; Figure 3b,d).

### 3.2 *Symbiodinium* community composition under excess DOC and DON

To assess the effects of excess DOC and DON on *Symbiodinium* community composition, we sequenced the ITS2 gene marker using next‐generation sequencing of 36 coral samples [3 coral replicates × 4 conditions (control vs. DOC; control vs. DON) × 3 time points] (Table S2). *Symbiodinium* communities of *P. verrucosa* were mainly composed of clades A and D in varying relative abundances, together accounting for >99% of all ITS2 sequences (Figure 2a). The remaining ITS2 sequences belonged to *Symbiodinium* of clades B and C, which consistently comprised <1% of all sequences. While excess DOC addition had no significant effect on *Symbiodinium* community composition over time (GLM, $\chi_{\left(5,n=18\right)}^{2}$ = 2.241, *p* = .815), observed decreases in the relative abundance of Clade D symbionts in controls might suggest that *Symbiodinium* communities were still adjusting to aquaria conditions. However, we did not observe such a shift in control fragments in the DON treatment. Control coral colonies in the excess DON experiment were stable, while under the excess DON treatment, they exhibited a significant shift in the relative clade abundance (GLM, $\chi_{\left(5,n=18\right)}^{2}$ = 18.703, *p* < .002) and *Symbiodinium* of Clade D almost entirely disappeared over time (Figure 2b).

### 3.3 Bacterial community composition under excess DOC and DON

To assess changes in seawater and coral‐associated bacterial communities, we sequenced 16S rRNA gene amplicon libraries of 72 samples, distributed over 36 coral fragments [3 coral replicates × 4 conditions (control vs. DOC; control vs. DON) × 3 time points] and 36 water samples [3 seawater aquaria replicates × 4 conditions (control vs. DOC; control vs. DON) × 3 time points]. After removal of unwanted sequences (i.e., sequences assigned to chloroplasts, mitochondria, archaea, eukaryotes, kit contaminants), a total of 3,576,201 sequences with an average length of 309 bp were retained for subsequent analyses (Table S3).

**Figure 4:**
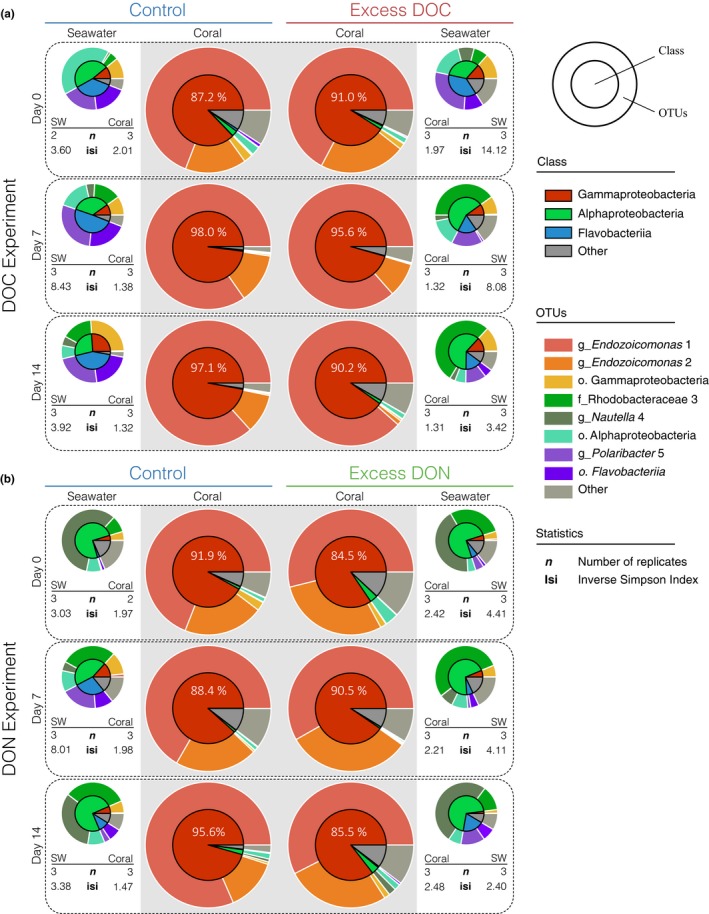
Bacterial community response in the coral *Pocillopora verrucos*a subjected to excess dissolved organic carbon (DOC) and excess dissolved organic nitrogen (DON) over time. (a) Excess DOC, (b) excess DON. Community composition is presented at the class and OTU level (inner vs. outer circle of pie charts, respectively). While seawater bacteria showed significant differences indicating effects of excess DOC and DON on community structure, coral‐associated bacterial communities remained remarkably stable over the course of the experiments (each pie plot represents means for *n* = 3). The two *Endozoicomonas* phylotypes (OTU 1 and OTU 2) constituted on average ~90% of all bacterial sequences

The dataset comprised 3,480 OTUs (at 97% similarity cutoff) of which 2,620 OTUs were present in seawater (1,354 of those were only found in seawater), 2,126 were associated with corals (860 of those were only found in corals), and 1,266 OTUs were present in seawater and corals (Table S4). Seawater and coral‐associated bacterial communities were significantly different from each other (AMOVA, *p *<* *.001; *F* = 53.571). Seawater bacterial community composition significantly changed over time and was different between experiments (AMOVA, *p *<* *.028, *F* = 4.134; Figure 4).

In contrast to the severe phenotypic response of the coral fragments as well as changes in the associated algal community, the associated bacterial community of *P. verrucosa* remained remarkably stable under excess DOC as well as under excess DON. We did not find statistically significant differences in coral bacterial community composition at the start of the experiment (AMOVA, *p *=* *.774, *F* = 0.059), nor did we find significant effects of time or treatment within the individual experiments (DOC: AMOVA, *p *=* *.172, *F* = 2.296; DON: AMOVA, *p *=* *.064, *F* = 5.542; Figure 4a,b). Even after the onset of coral bleaching (in the case of excess DOC) and at the point of host mortality at day 14 (>90% tissue loss, in the case of excess DON), the bacterial community remained largely invariant in its overall structure, and no significant changes in bacterial alpha diversity indices (Inverse Simpson Index, Simpson's Evenness) were observed (Figure 3; DOC: ANOVA; *p *=* *.82, *F* = 0.054 and *p *=* *.90, *F* = 0.015; DON: ANOVA: *p *=* *.097, *F* = 3.165 and *p *=* *.17, *F* = 2.063; for full overview, see Table S3).

Bacterial community composition was highly uneven and dominated by only two bacterial OTUs of the genus *Endozoicomonas* (Oceanospirillales: Hahellaceae, recently put into the proposed family Endozoicomonadaceae (Bartz, Blom, Busse, Mvie, Hardt et al., 2017)) with a combined relative average abundance of 90% across all coral fragments. These two OTUs also comprised the “core” microbiome, defined as OTUs present in 100% of all coral samples (for a complete presentation of coral‐associated OTU abundances, see Table S4). Notably, after omitting all *Endozoicomonas* sequences from analysis, no treatment effects on the remaining coral‐associated bacterial communities were apparent under excess DOC (AMOVA, *p *=* *.843, *F* = −0.006) or excess DON (AMOVA, *p *=* *.894, *F* = −0.998). However, despite the overall stable bacterial community and the dominant contribution of *Endozoicomonas* OTUs to the overall bacterial community composition, changes in the relative abundance of the two *Endozoicomonas* OTUs became apparent. *Endozoicomonas* OTU 2 decreased substantially under excess DOC from 22% to 1% within 14 days of treatment (corresponding to an increase in relative contribution of *Endozoicomonas* OTU 1 from 67% to 89%). In contrast, *Endozoicomonas* OTU 1 and OTU 2 remained proportionally stable under excess DON over 14 days (about 56% for OTU 1 vs. 29% for OTU 2 average relative contribution). The reasons for this are unclear at present, although metabolic subfunctionalization/specialization of *Endozoicomonas* taxa residing in corals was suggested previously (Neave, Michell, Apprill, & Voolstra, 2017; Neave, Rachmawati, et al., 2017).

## 4 DISCUSSION

To assess the stability and flexibility of coral holobiont structure in the common reef‐building coral *P. verrucosa*, we monitored community composition of *Symbiodinium* and bacteria to severely altered nutrient conditions. Excess DOC and DON clearly impaired coral health and functioning within days as indicated by coral bleaching and tissue loss (sloughing) and eventually mortality, respectively. Despite the severe phenotypic responses, coral‐associated bacterial communities, in contrast to *Symbiodinium* communities, remained remarkably stable and were dominated by two OTUs of the genus *Endozoicomonas*, comprising on average 90% of the coral‐associated 16S rRNA gene amplicon sequences. This outcome has implications for our understanding of coral holobiont structure and microbiome flexibility and stability, as discussed in the following.

### 4.1 Breakdown of the coral‐algae symbiosis

Both DOC and DON treatments affected the coral‐algae symbiosis, although the responses of *Symbiodinium* and coral stress phenotypes differed between both experimental treatments. While under excess DOC corals experienced a rapid loss of *Symbiodinium* (for a detailed discussion see Pogoreutz, Rädecker, Cárdenas, Gärdes, Wild, et al., 2017), the *Symbiodinium* population density and chlorophyll *a* content rapidly increased under elevated DON as suggested by our measurements and the visibly darkened tissue, which constitutes a common response of coral holobionts to elevated nitrogen levels (Ezzat, Maguer, Grover, & Ferrier‐Pagès, 2015; Falkowski et al., 1984, 1993).

The rapid proliferation of algal symbionts under excess DON was associated with fluctuations in the abundance of the two dominant symbiont clades A and D (as opposed to a stable algal community composition under excess DOC). No symbiont shuffling was observed (Mieog, Van Oppen, Cantin, Stam, & Olsen, 2007). Instead, *P. verrucosa* exhibited strong clade fidelity concomitant with an increase in clade A *Symbiodinium*. Hence, clade A symbionts seem to perform better in high nitrogen environments when compared to symbionts from clade D. In this regard, it is interesting to note that clade D *Symbiodinium* exhibited an inferior capacity for nitrogen acquisition compared to other clades at ambient temperatures in previous work (Baker, Andras, Jordán‐Garza, & Fogel, 2013; Pernice et al., 2015).

At large, our findings may explain the prevalence of clade A symbionts in the highly phototrophic *P. verrucosa* along most of the Red Sea basin (Sawall et al., 2015). Importantly, clade A symbionts are rare in most scleractinian corals (LaJeunesse et al., 2004) and are often considered opportunistic (Stat, Morris, & Gates, 2008). In the oligotrophic conditions of the Red Sea, however, the association with clade A *Symbiodinium* may increase holobiont productivity due to its assumingly efficient nitrogen uptake capability (Aranda et al., 2016). Under excess DON, on the other hand, we argue that clade A *Symbiodinium* may be detrimental to *P. verrucosa* holobiont health due to opportunistic growth and reduced carbon translocation.

### 4.2 Stable dominance of *Endozoicomonas* during bleaching and mortality

Despite the severe coral stress responses, the bacterial community structure of *P. verrucosa* remained dominated by *Endozoicomonas* under excess DOC and DON over the 14‐day experimental treatments. This observation does not rule out that community shifts occurred within the rare fraction of the microbiome, but the bacterial community at large was consistent. Importantly, we did not observe an intrusion and propagation of putative opportunistic or pathogenic bacteria from the surrounding seawater, which harbored bacterial communities that were highly distinct from bacteria associated with *P. verrucosa* (Figure 4). While the absence of bacterial community changes in coral holobionts counters previous work (Vega Thurber et al., 2009, 2012; Ziegler et al., 2016), recent work from the Red Sea reports on similarly stable bacterial communities in *P. verrucosa* across sites subject to differential anthropogenic impact (sewage, municipal waste water, and sediment input). Notably, this bacterial community “stability” in *P. verrucosa* as reported by Ziegler et al. (2016) could largely be attributed to the high abundance of taxa in the family “Endozoicimonaceae”. Similarly, in our current study the stable bacterial community of *P. verrucosa* was driven by the prevalence and dominance of two *Endozoicomonas* OTUs. Importantly, the same two *Endozoicomonas* phylotypes were previously shown to prevail in the microbiome of *P. verrucosa* across its entire global distribution range with little geographic partitioning, suggesting a particularly intimate and conserved host‐microbe relationship (Neave, Apprill, Ferrier‐Pagès, & Voolstra, 2016).

*Endozoicomonas* are Gammaproteobacteria frequently observed in reef‐building corals (Bayer et al., 2013; Bourne et al., 2008; Glasl, Herndl, & Frade, 2016; Neave, Michell, et al., 2017; Neave, Rachmawati, et al., 2017; Pantos, Bongaerts, Dennis, Tyson, & Hoegh‐Guldberg, 2015; Pogoreutz, Rädecker, Cárdenas, Gärdes, Wild, et al., 2017) and highly dominant in pocilloporid hosts where they were found to reside deep within the gastrovascular tissues (Bayer et al., 2013; Neave, Rachmawati, et al., 2017). More generally, *Endozoicomonas* are commonly assumed to provide an important role in coral holobiont functioning due to their widespread prevalence and high abundance in many coral species (Bayer et al., 2013; Gignoux‐Wolfsohn, Aronson, & Vollmer, 2017; Glasl et al., 2016; Jessen et al., 2013; Meyer, Gunasekera, Scott, Paul, & Teplitski, 2016; Neave et al., 2016; Neave, Rachmawati, et al., 2017) and apparent metabolic versatility (Ding, Shiu, Chen, Chiang, & Tang, 2016; Hyun et al., 2014; Neave, Michell, et al., 2017; Yang et al., 2010). Further, reductions in the abundance of *Endozoicomonas* in stressed, diseased, or bleached corals are reported in a number of studies, suggesting that pervasive abundance of *Endozoicomonas* might be an indicator of habitat suitability (Bourne et al., 2008; Cárdenas et al., 2012; Gignoux‐Wolfsohn et al., 2017; Meyer et al., 2016; Morrow et al., 2014; Roder et al., 2015; Röthig et al., 2016; Ziegler et al., 2016). This is in contrast to our findings here, where we see a quasi‐invariant association with *Endozoicomonas* that makes up on average 90% of the bacterial community, despite the severe nutrient treatments and distinct stress phenotypes, (coral bleaching and progressed tissue sloughing, respectively). Hence, our data suggest a near‐obligate association, much like the association with *Symbiodinium*, although the specific functional importance of this relationship still needs to be defined (Neave et al., 2016; Neave, Michell, et al., 2017).

### 4.3 Structural stability of bacterial communities: implications for holobiont functioning

The remarkable stability of the association between *Endozoicomonas* and *P. verrucosa* extends our understanding of coral holobiont structure and composition. In contrast to recent studies that suggest a flexibility of corals to associate with different bacteria under adverse environmental conditions (Röthig, Roik, Yum, & Voolstra, 2017; Röthig et al., 2016; Ziegler, Seneca, et al., 2017), our work provides evidence for a coral holobiont with a structurally stable bacterial microbiome even under host mortality. While it is possible that adaptive or opportunistic shifts may have occurred for rare members of the microbiome, the structural abundance of dominant microbiome members appears to be maintained even under adverse environmental conditions. This suggests different levels of microbiome structural flexibility across coral species. Coral holobionts may be distributed along a continuum ranging from structural adaptability to stability of their bacterial microbiomes, that is, different structural landscapes of holobionts may exist. Consequently, coral holobionts with adaptable bacterial communities may respond rather dynamically and readily to environmental change, as observed in Fungiidae (Roder et al., 2015; Röthig et al., 2016), Acroporidae (Ziegler et al., 2016; Ziegler, Seneca, et al., 2017), or Dendrophyllidae (Röthig et al., 2017). By comparison, coral hosts with structurally “stable” (and presumably strongly selected) microbiomes may host highly uneven bacterial communities (as observed in *P. verrucosa*) with a potentially very specialized set of (metabolic) functions (Ley, Peterson, & Gordon, 2006; Sawall et al., 2015; Ziegler et al., 2014, 2016). Such strongly structured bacterial communities may provide an advantage under highly stable conditions, but at the same time may implicitly come with the restriction to a comparatively narrow ecological niche space, as known for heritable obligate host‐microbe symbioses (Bennett & Moran, 2015). Further, the strong reliance on few selected bacterial symbionts may come at the cost of low stress resistance under adverse environmental conditions (Wittebolle et al., 2009). Indeed, Red Sea *P. verrucosa* holobionts can be considered highly specialized as indicated by their fairly limited bathymetric range and restriction to shallow sunlit and nutrient‐poor waters, where fairly stable environmental conditions prevail (Pogoreutz, Rädecker, Cárdenas, Gärdes, Voolstra, et al., 2017; Sawall et al., 2015; Ziegler et al., 2014).

Future studies should assess coral microbial community association and flexibility under environmental stress or across environmental gradients including coral species covering different ecological traits, for example, autotrophy versus heterotrophy or spawning versus brooding corals. This will further help to understand whether coral species indeed represent different holobiont structural landscapes (i.e., specialized and stable vs. functionally redundant and flexible) and how bacterial community stability aligns with environmental resilience.

## DATA ACCESSIBILITY

Microbial data as well as aquaria seawater conditions are available as Supporting Information. Flow cytometry data of *Symbiodnium* communities are available from the Dryad Digital Repository: https://doi.org/10.5061/dryad.23gp4. Raw sequence data determined in this study are available at NCBI under BioProject Accession No. PRJNA394597 (https://www.ncbi.nlm.nih.gov/bioproject/PRJNA394597). Abundant coral bacterial microbiome OTU reference sequences are available under GenBank Accession numbers MG725756 ‐ MG725813 (https://www.ncbi.nlm.nih.gov/nuccore/?term=MG725756:MG725813[accn]).

## CONFLICT OF INTEREST

None declared.

## AUTHORS CONTRIBUTION

CP, NR, CW, CRV designed and conceived the study; CP, NR, AC generated data; CP, NR, CRV analyzed data; AG, CW, CRV contributed reagents/tools/materials; CP, NR, CRV wrote the manuscript; all authors read and approved the final manuscript.
