# Supplementary material for: Dominance of Endozoicomonas bacteria throughout coral bleaching and mortality suggests structural inflexibility of the Pocillopora verrucosa microbiome
Source: Ecol Evol. 2018 Jan 25;8(4):2240–52. doi: 10.1002/ece3.3830 (PMC5817147; doi:10.1002/ece3.3830)
Supplement: Supplementary file 1 [file ECE3-8-2240-s001.pdf]

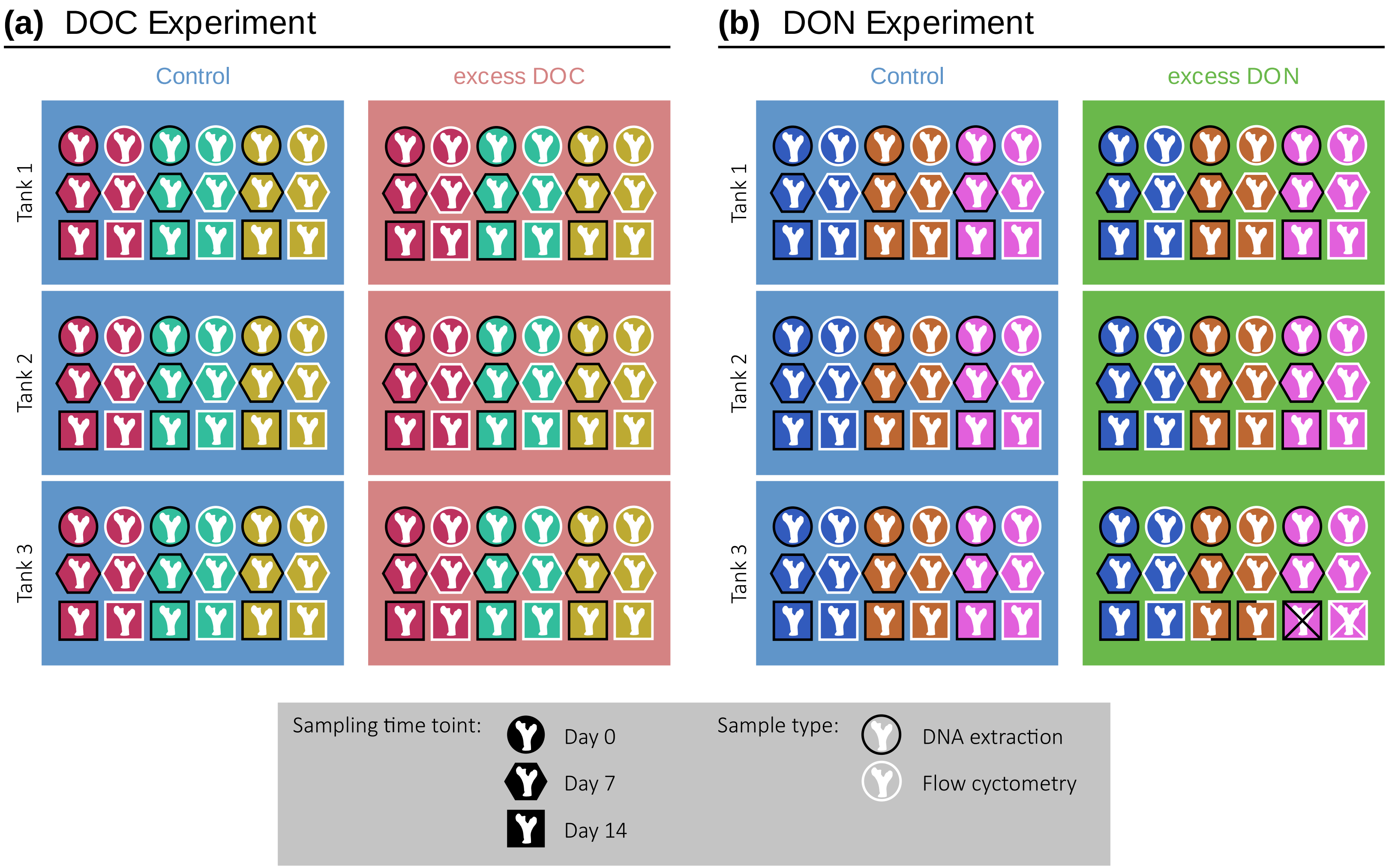

Supplementary Figure 1. Overview of sampling scheme of coral colonies during the (a) DOC and (b) DON experiments. Color of fragments denotes the colony of origin. Shape of fragment denotes time point of sampling. The outline color of fragments denotes the type of analysis that the sampled fragments were used for. Crossed out fragments were not available for analysis due to mortality.
