# Supplementary material for: Dominance of Endozoicomonas bacteria throughout coral bleaching and mortality suggests structural inflexibility of the Pocillopora verrucosa microbiome
Source: Ecol Evol. 2018 Jan 25;8(4):2240–52. doi: 10.1002/ece3.3830 (PMC5817147; doi:10.1002/ece3.3830)
Supplement: Supplementary file 2 [file ECE3-8-2240-s002.docx]

**Supplementary Table 1.** Maintenance conditions in seawater tanks for excess dissolved organic carbon (DOC) and excess dissolved organic nitrogen (DON) experiments over time. Values are presented as mean ± SE. CON = control; TDN = total dissolved nitrogen content.

| **Experiment** | **Day** | **Treatment** | **Temperature [T°C]** | **Salinity** | **DOC [µM]** | **TDN [µM]** |
| --- | --- | --- | --- | --- | --- | --- |
| excess DOC | 0 | CON | 26.8 ± 0.0 | 41.6 ± 0.0 | 206 ± 9 |  |
|  |  | DOC | 26.9 ± 0.0 | 42.0 ± 0.0 | 154 ± 3 |  |
|  | 7 | CON | 26.8 ± 0.0 | 40.9 ± 0.0 | 144 ± 8 |  |
|  |  | DOC | 26.8 ± 0.0 | 40.7 ± 0.0 | **868 ± 7** |  |
|  | 14 | CON | 26.8 ± 0.0 | 40.9 ± 0.0 | 130 ± 1 |  |
|  |  | DOC | 26.9 ± 0.0 | 40.7 ± 0.0 | **1471 ± 7** |  |
| excess DON | 0 | CON | 25.9 ± 0.1 | 40.8 ± 0.1 |  | 16.8 ± 2.7 |
|  |  | DON | 26.1 ± 0.1 | 40.9 ± 0.1 |  | 16.5 ± 0.4 |
|  | 7 | CON | 26.5 ± 0.2 | 40.9 ±0.1 |  | 13.0 ± 0.8 |
|  |  | DON | 26.8 ± 0.1 | 40.6 ± 0.2 |  | **589.8 ± 1.6** |
|  | 14 | CON | 26.5 ± 0.2 | 40.5 ± 0.2 |  | 10.7 ± 0.1 |
|  |  | DON | 26.9 ± 0.1 | 40.5 ± 0.2 |  | **793.1 ± 3.6** |
