# Supplementary material for: Dominance of Endozoicomonas bacteria throughout coral bleaching and mortality suggests structural inflexibility of the Pocillopora verrucosa microbiome
Source: Ecol Evol. 2018 Jan 25;8(4):2240–52. doi: 10.1002/ece3.3830 (PMC5817147; doi:10.1002/ece3.3830)
Supplement: Supplementary file 3 [file ECE3-8-2240-s003.docx]

**Supplementary Table 2.** ITS2 marker gene sequencing of *Symbiodinium* populations associated with the coral *Pocillopora verrucosa* under excess dissolved organic carbon (DOC) and excess dissolved organic nitrogen (DON). Shown are the total number of sequences (Sum) and the distribution of sequences over clades for the different time points and treatment groups. C = control; N = excess nutrient treatment; d = days.

|  | |  |  | |  | | **ITS2 sequences** | | | | | |
| --- | --- | --- | --- | --- | --- | --- | --- | --- | --- | --- | --- | --- |
| Experiment | | Group |  | | group | | Sum | | Clade A | Clade B | Clade C | Clade D |
| DOC | | Coral | Control 0d | | DOC_coral_C0_1 | | 193950 | | 193852 | 0 | 50 | 48 |
|  | |  |  | | DOC_coral_C0_2 | | 110791 | | 14499 | 0 | 57 | 96235 |
|  | |  |  | | DOC_coral_C0_3 | | 244085 | | 85139 | 0 | 0 | 158946 |
|  | |  | Control 7d | | DOC_coral_C1_1 | | 5618 | | 5124 | 0 | 0 | 494 |
|  | |  |  | | DOC_coral_C1_2 | | 76721 | | 76578 | 0 | 0 | 143 |
|  | |  |  | | DOC_coral_C1_3 | | 65038 | | 43265 | 0 | 0 | 21773 |
|  | |  | Control 14d | | DOC_coral_C2_1 | | 107789 | | 107750 | 4 | 2 | 33 |
|  | |  |  | | DOC_coral_C2_2 | | 44081 | | 43745 | 59 | 143 | 134 |
|  | |  |  | | DOC_coral_C2_3 | | 201143 | | 132618 | 11 | 17 | 68497 |
|  | |  | DOC 0d | | DOC_coral_N0_1 | | 125778 | | 11732 | 0 | 1 | 114045 |
|  | |  |  | | DOC_coral_N0_2 | | 144517 | | 144465 | 0 | 0 | 52 |
|  | |  |  | | DOC_coral_N0_3 | | 132509 | | 122417 | 38 | 305 | 9749 |
|  | |  | DOC 7d | | DOC_coral_N1_1 | | 105359 | | 105112 | 162 | 23 | 62 |
|  | |  |  | | DOC_coral_N1_2 | | 188753 | | 136565 | 0 | 0 | 52188 |
|  | |  |  | | DOC_coral_N1_3 | | 196358 | | 122283 | 0 | 60 | 74015 |
|  | |  | DOC 14d | | DOC_coral_N2_1 | | 187 | | 148 | 0 | 0 | 39 |
|  | |  |  | | DOC_coral_N2_2 | | 135656 | | 135512 | 22 | 49 | 73 |
|  | |  |  | | DOC_coral_N2_3 | | 164659 | | 69874 | 18 | 12 | 94755 |
| DON | |  |  | |  | |  | |  |  |  |  |
|  | | Coral | Control 0d | | DON_coral_C0_1 | | 73982 | | 73024 | 0 | 0 | 958 |
|  | |  |  | | DON_coral_C0_2 | | 36984 | | 5600 | 0 | 0 | 31384 |
|  | |  |  | | DON_coral_C0_3 | | 11859 | | 11477 | 0 | 0 | 382 |
|  | |  | Control 7d | | DON_coral_C1_1 | | 62763 | | 14256 | 0 | 0 | 48507 |
|  | |  |  | | DON_coral_C1_2 | | 67975 | | 63934 | 0 | 0 | 4041 |
|  | |  |  | | DON_coral_C1_3 | | 58478 | | 56318 | 0 | 122 | 2038 |
|  | |  | Control 14d | | DON_coral_C2_1 | | 70889 | | 42542 | 0 | 0 | 28347 |
|  | |  |  | | DON_coral_C2_2 | | 63703 | | 22863 | 0 | 0 | 40840 |
|  | |  |  | | DON_coral_C2_3 | | 59245 | | 59230 | 0 | 0 | 15 |
|  | |  | DON 0d | | DON_coral_N0_1 | | 54408 | | 54395 | 0 | 0 | 13 |
|  | |  |  | | DON_coral_N0_2 | | 69526 | | 56771 | 0 | 0 | 12755 |
|  | |  |  | | DON_coral_N0_3 | | 38938 | | 31242 | 0 | 90 | 7606 |
|  | |  | DON 7d | | DON_coral_N1_1 | | 43578 | | 43564 | 0 | 0 | 14 |
|  | |  |  | | DON_coral_N1_2 | | 59990 | | 55725 | 0 | 47 | 4218 |
|  | |  |  | | DON_coral_N1_3 | | 76030 | | 75947 | 0 | 0 | 83 |
|  | |  | DON 14d | | DON_coral_N2_1 | | 82629 | | 81970 | 0 | 0 | 659 |
|  | |  |  | | DON_coral_N2_2 | | 91530 | | 91351 | 0 | 0 | 179 |
|  | |  |  | | DON_coral_N2_3 | | 57839 | | 57826 | 0 | 0 | 13 |
|  |  | |  |  | |  | |  |  |  |  |  |
|  |  | |  |  | |  | |  |  |  |  |  |
|  |  | |  |  | |  | |  |  |  |  |  |
