# Supplementary material for: Dominance of Endozoicomonas bacteria throughout coral bleaching and mortality suggests structural inflexibility of the Pocillopora verrucosa microbiome
Source: Ecol Evol. 2018 Jan 25;8(4):2240–52. doi: 10.1002/ece3.3830 (PMC5817147; doi:10.1002/ece3.3830)
Supplement: Supplementary file 4 [file ECE3-8-2240-s004.docx]

**Supplementary Table 3.** Statistics of 16S rRNA gene amplicon sequencing and diversity indices of bacterial populations associated with seawater and the coral *Pocillopora verrucosa* under excess dissolved organic carbon (DOC) and excess dissolved organic nitrogen (DON). Shown is the distribution of sequences over samples and time points. Total number of OTUs = 3,480 OTUs (at 97% similarity cutoff). C = control; N = excess nutrient treatment; SW = seawater; d = days; nseqs = number of sequences.

|  |  |  |  | **16S rRNA gene** | | | | | |
| --- | --- | --- | --- | --- | --- | --- | --- | --- | --- |
|  |  |  |  |  | Diversity Indices | | |  |  |
| Experiment | Group |  | group | nseqs | chao | invsimpson | simpsoneven | coverage |  |
| DOC | Seawater | Control 0d | DOC_SW_C0_1 | 16433 | 230.00 | 4.05 | 0.03 | 1.00 |  |
|  |  |  | DOC_SW_C0_3 | 25107 | 648.11 | 3.15 | 0.01 | 0.99 |  |
|  |  | Control 7d | DOC_SW_C1_1 | 16600 | 301.71 | 3.51 | 0.02 | 0.99 |  |
|  |  |  | DOC_SW_C1_2 | 16562 | 214.65 | 3.94 | 0.03 | 1.00 |  |
|  |  |  | DOC_SW_C1_3 | 25346 | 731.90 | 17.84 | 0.03 | 0.99 |  |
|  |  | Control 14d | DOC_SW_C2_1 | 19407 | 301.06 | 4.46 | 0.02 | 1.00 |  |
|  |  |  | DOC_SW_C2_2 | 16148 | 359.18 | 3.09 | 0.01 | 0.99 |  |
|  |  |  | DOC_SW_C2_3 | 19544 | 315.32 | 4.21 | 0.02 | 1.00 |  |
|  |  | DOC 0d | DOC_SW_N0_1 | 11566 | 322.21 | 5.29 | 0.03 | 0.99 |  |
|  |  |  | DOC_SW_N0_2 | 23517 | 420.00 | 3.71 | 0.01 | 1.00 |  |
|  |  |  | DOC_SW_N0_3 | 18275 | 842.62 | 33.35 | 0.05 | 0.99 |  |
|  |  | DOC 7d | DOC_SW_N1_1 | 34244 | 538.02 | 2.92 | 0.01 | 1.00 |  |
|  |  |  | DOC_SW_N1_2 | 23666 | 420.00 | 2.24 | 0.01 | 1.00 |  |
|  |  |  | DOC_SW_N1_3 | 41167 | 848.48 | 19.07 | 0.03 | 1.00 |  |
|  |  | DOC 14d | DOC_SW_N2_1 | 23353 | 484.52 | 2.41 | 0.01 | 0.99 |  |
|  |  |  | DOC_SW_N2_2 | 52675 | 847.51 | 4.92 | 0.01 | 1.00 |  |
|  |  |  | DOC_SW_N2_3 | 67928 | 804.01 | 2.94 | 0.01 | 1.00 |  |
|  | Coral | Control 0d | DOC_coral_C0_1 | 63156 | 787.08 | 2.16 | 0.00 | 1.00 |  |
|  |  |  | DOC_coral_C0_2 | 72932 | 624.13 | 2.37 | 0.01 | 1.00 |  |
|  |  |  | DOC_coral_C0_3 | 152464 | 283.00 | 1.52 | 0.01 | 1.00 |  |
|  |  | Control 7d | DOC_coral_C1_1 | 128279 | 377.12 | 1.30 | 0.01 | 1.00 |  |
|  |  |  | DOC_coral_C1_2 | 122351 | 727.31 | 1.72 | 0.00 | 1.00 |  |
|  |  |  | DOC_coral_C1_3 | 86375 | 104.63 | 1.12 | 0.01 | 1.00 |  |
|  |  | Control 14d | DOC_coral_C2_1 | 83379 | 501.29 | 1.16 | 0.00 | 1.00 |  |
|  |  |  | DOC_coral_C2_2 | 65931 | 381.28 | 1.57 | 0.01 | 1.00 |  |
|  |  |  | DOC_coral_C2_3 | 119880 | 434.00 | 1.22 | 0.00 | 1.00 |  |
|  |  | DOC 0d | DOC_coral_N0_1 | 119450 | 600.12 | 2.18 | 0.00 | 1.00 |  |
|  |  |  | DOC_coral_N0_2 | 50941 | 475.02 | 2.25 | 0.01 | 1.00 |  |
|  |  |  | DOC_coral_N0_3 | 163714 | 600.62 | 1.46 | 0.00 | 1.00 |  |
|  |  | DOC 7d | DOC_coral_N1_1 | 101299 | 117.38 | 1.44 | 0.02 | 1.00 |  |
|  |  |  | DOC_coral_N1_2 | 238017 | 243.86 | 1.13 | 0.01 | 1.00 |  |
|  |  |  | DOC_coral_N1_3 | 122294 | 376.40 | 1.40 | 0.01 | 1.00 |  |
|  |  | DOC 14d | DOC_coral_N2_1 | 123381 | 379.11 | 1.06 | 0.00 | 1.00 |  |
|  |  |  | DOC_coral_N2_2 | 80523 | 389.41 | 1.65 | 0.01 | 1.00 |  |
|  |  |  | DOC_coral_N2_3 | 189852 | 546.90 | 1.21 | 0.00 | 1.00 |  |
| DON |  |  |  |  |  |  |  |  |  |
|  | Seawater | Control 0d | DON_SW_C0_1 | 29680 | 593.43 | 2.07 | 0.00 | 0.99 |  |
|  |  |  | DON_SW_C0_2 | 23399 | 492.28 | 2.06 | 0.01 | 0.99 |  |
|  |  |  | DON_SW_C0_3 | 28278 | 752.48 | 4.96 | 0.01 | 0.99 |  |
|  |  | Control 7d | DON_SW_C1_1 | 10470 | 358.38 | 3.71 | 0.02 | 0.99 |  |
|  |  |  | DON_SW_C1_2 | 14489 | 660.28 | 15.30 | 0.03 | 0.99 |  |
|  |  |  | DON_SW_C1_3 | 14854 | 678.22 | 5.01 | 0.01 | 0.99 |  |
|  |  | Control 14d | DON_SW_C2_1 | 24828 | 364.50 | 2.66 | 0.01 | 1.00 |  |
|  |  |  | DON_SW_C2_2 | 43002 | 696.92 | 5.48 | 0.01 | 1.00 |  |
|  |  |  | DON_SW_C2_3 | 47036 | 238.50 | 1.99 | 0.01 | 1.00 |  |
|  |  |  | DON_SW_N0_1 | 31166 | 482.46 | 2.93 | 0.01 | 1.00 |  |
|  |  |  | DON_SW_N0_2 | 26891 | 306.43 | 2.54 | 0.01 | 1.00 |  |
|  |  |  | DON_SW_N0_3 | 25134 | 691.09 | 7.76 | 0.01 | 0.99 |  |
|  |  |  | DON_SW_N1_1 | 27203 | 622.64 | 1.97 | 0.00 | 0.99 |  |
|  |  |  | DON_SW_N1_2 | 18310 | 670.86 | 7.72 | 0.01 | 0.99 |  |
|  |  |  | DON_SW_N1_3 | 22164 | 309.61 | 2.64 | 0.01 | 1.00 |  |
|  |  |  | DON_SW_N2_1 | 18900 | 221.45 | 1.57 | 0.01 | 1.00 |  |
|  |  |  | DON_SW_N2_2 | 16632 | 262.65 | 3.68 | 0.02 | 1.00 |  |
|  |  |  | DON_SW_N2_3 | 34064 | 201.50 | 1.94 | 0.01 | 1.00 |  |
|  | Coral | Control 0d | DON_coral_C0_1 | 32332 | 273.02 | 3.00 | 0.01 | 1.00 |  |
|  |  |  | DON_coral_C0_2 | 37527 | 224.53 | 1.42 | 0.01 | 1.00 |  |
|  |  |  | DON_coral_C0_3 | 35239 | 224.63 | 1.48 | 0.01 | 1.00 |  |
|  |  | Control 7d | DON_coral_C1_1 | 19934 | 64.27 | 1.79 | 0.04 | 1.00 |  |
|  |  |  | DON_coral_C1_2 | 62203 | 388.16 | 1.59 | 0.00 | 1.00 |  |
|  |  |  | DON_coral_C1_3 | 46350 | 107.94 | 2.55 | 0.03 | 1.00 |  |
|  |  | Control 14d | DON_coral_C2_1 | 22957 | 199.66 | 1.88 | 0.01 | 1.00 |  |
|  |  |  | DON_coral_C2_2 | 35112 | 344.44 | 1.34 | 0.00 | 1.00 |  |
|  |  |  | DON_coral_C2_3 | 23268 | 91.94 | 1.19 | 0.02 | 1.00 |  |
|  |  | DON 0d | DON_coral_N0_1 | 23868 | 73.21 | 2.00 | 0.03 | 1.00 |  |
|  |  |  | DON_coral_N0_2 | 36422 | 279.12 | 1.75 | 0.01 | 1.00 |  |
|  |  |  | DON_coral_N0_3 | 33879 | 96.45 | 3.52 | 0.04 | 1.00 |  |
|  |  | DON 7d | DON_coral_N1_1 | 29839 | 73.09 | 1.59 | 0.03 | 1.00 |  |
|  |  |  | DON_coral_N1_2 | 23648 | 145.13 | 2.20 | 0.02 | 1.00 |  |
|  |  |  | DON_coral_N1_3 | 29892 | 88.50 | 2.83 | 0.04 | 1.00 |  |
|  |  | DON 14d | DON_coral_N2_1 | 24533 | 64.27 | 1.65 | 0.04 | 1.00 |  |
|  |  |  | DON_coral_N2_2 | 34438 | 391.59 | 3.11 | 0.01 | 1.00 |  |
|  |  |  | DON_coral_N2_3 | 32504 | 488.25 | 2.70 | 0.01 | 1.00 |  |
